# Interstitial Cells of Blood Vessels

**DOI:** 10.1100/tsw.2010.123

**Published:** 2010-06-16

**Authors:** Vladimír Pucovský

**Affiliations:** Centre for Vision and Vascular Science, School of Medicine, Dentistry and Biomedical Sciences, Queen's University Belfast, UK

**Keywords:** interstitial cell, vascular smooth muscle cell, phenotype, intracellular calcium, filopodia, confocal imaging, immunocytochemistry, patch clamp

## Abstract

Blood vessels are made up of several distinct cell types. Although it was originally thought that the tunica media of blood vessels was composed of a homogeneous population of fully differentiated smooth muscle cells, more recent data suggest the existence of multiple smooth muscle cell subpopulations in the vascular wall. One of the cell types contributing to this heterogeneity is the novel, irregularly shaped, noncontractile cell with thin processes, termed *interstitial cell*, found in the tunica media of both veins and arteries. While the principal role of interstitial cells in veins seems to be pacemaking, the role of arterial interstitial cells is less clear. This review summarises the knowledge of the functional and structural properties of vascular interstitial cells accumulated so far, offers hypotheses on their physiological role, and proposes directions for future research.

## INTRODUCTION

Blood vessels are made up of several distinct cell types, such as endothelial cells, pericytes, smooth muscle cells, neurones (endings), fibroblasts, and various inflammatory cells, which all work in concert to ensure an optimal supply of nutrients and oxygen to tissues. Until approximately the early 1990s, the prevailing view was that the muscular layer, *tunica media*, consisted of a homogeneous population of mature, differentiated, contractile, vascular smooth muscle cells (VSMCs). This was despite the occasional paper which demonstrated the presence of cells with processes in various tissues, including blood vessels[1,2]. At that time, the cells with processes were thought to be of neuronal nature, so they received little attention. A number of works starting from the 1990s demonstrated the presence of distinct VSMC subpopulations in normal blood vessels[3,4,5,6,7]. Progress in cell isolation and microscopy techniques over the last several decades has made it possible to observe live single cells in unprecedented detail. In the VSMC field in the early 2000s, this ability has allowed, among other discoveries, the (re)discovery of rare, irregularly shaped, noncontractile cells with thin processes, accompanying VSMCs and their functional and structural investigation on a single cell level.

The presence of cells which fall in the category of irregularly shaped with processes have so far been confirmed in every tissue which contains smooth muscle cells, apart from airway smooth muscle (oesophagus[8,9], stomach[10,11], small intestine[12,13], colon[14,15], ureter[16,17], urinary bladder[18,19,20,21,22], urethra[23,24,25,26,27], arteries[28,29,30,31], veins[32,33,34,35], lymphatic vessels[36], uterus[37,38], Fallopian tubes[39], prostate[40,41], and vas deferens[42]). Cells of similar morphology have also been described in the heart muscle[43], close to cardiomyocytes, in both atria[44,45] and ventricles[46], suggesting their presence throughout the cardiovascular system. Earlier hypotheses that irregularly shaped cells with processes accompany smooth muscle cells throughout the body may need extending, as these cells were also reported in locations unrelated to and/or at some distance from muscle[47,48,49,50].

As is usually the case in new and developing areas, there is a considerable dissonance in the nomenclature of these cells (to which the author contributed with a fair share). This multitude of terms will yet have to result in a scientific community–wide agreement on the name and (preliminary) classification of all the "strange" cells with processes in blood vessels and other tissues (apart from the well-established *interstitial cells of Cajal* [ICCs] in the gastrointestinal tract). These cells have so far been referred to as ICCs[18,31,32,33,34,35,48,51], ICC-like cells[28,52], immature VSMCs[29], interstitial cells[30,53,54,55], pacemaker cells[23,56], interstitial Cajal-like cells[16,39,44,46,47,49,50,57] or, most recently, telocytes[58,59]. From the research so far, it is already clear that, rather than a uniform group, these cells are a conglomerate of subpopulations, each with its slightly different structure and function, depending on their localisation in the body. In some instances, the naming of these cells as ICCs seems premature, as not enough data have been collected about them to satisfy the ultrastructural[60,61] or functional criteria for an ICC. Until we gain a better understanding of their origin and of the differences between their subpopulations, a single name for them would help to avoid much of the confusion in future. Similar to Harhun et al.[54], these cells will be referred to in this text as *interstitial cells (ICs)*, which is a compromise between inclusiveness of all subpopulations and accuracy in naming the phenomenon.

This review will be restricted to vascular ICs. Gastrointestinal tract ICs, known as ICCs, have been the subject of extensive investigation over a number of years, which resulted in a wealth of data and a number of review papers (e.g., [62,63,64,65]). Likewise, there have been several review papers published recently on urinary tract ICs[16,66,67,68,69]. As for the ICs in other locations, their investigation is at the very early stage and data, especially on their function, are scarce and require further studies. This review builds on two earlier reviews[53,54], summarises the knowledge of the structure and functional properties of vascular ICs accumulated since their rediscovery 7 years ago, offers hypotheses on their physiological role, and proposes directions for future research and potential utilisation.

## MORPHOLOGY

ICs in blood vessels are irregularly shaped with long thin processes, filopodia, which are their most prominent feature (Fig. 1). The thickness of filopodia ranges from a fraction to a couple of micrometers, and their length can be up to a couple of hundred micrometers[35]. In the majority of cases, the filopodia do not have varicosities (moniliform filopodia were described only for ICs of rat mesentery[50]) and are sometimes seen branching (up to $5^{th}$ order[35]). Confocal imaging of calcium in cells loaded with a calcium-sensitive fluorescent indicator[32], and electron microscopy of ICs either in single cell suspension or in intact tissue[28], have confirmed that the filopodia are continuous with the cell body.

The function of filopodia was investigated in more detail in ICs of guinea pig small mesenteric arteries. It was found that in single isolated ICs, they were growing in length (max. speed at 20–22°C of ~800 nm/min[28]) in the absence of serum or nutrients other than glucose. The elongation of filopodia seemed random in direction. They were observed growing sometimes toward the neighbouring VSMCs and sometimes past them. The ability of latrunculin B, an actin polymerisation inhibitor, to abolish the growth of filopodia suggested that their elongation is based on actin polymerisation[28]. It is still not clear what role the elongation of filopodia plays in ICs and whether it occurs in the intact tissue as well. It has not been reported for ICs in any other tissues. So far, it is still only a hypothesis that the elongation of filopodia is a reaction of ICs to the loss of contact with the extracellular matrix (ECM) and the adjoining

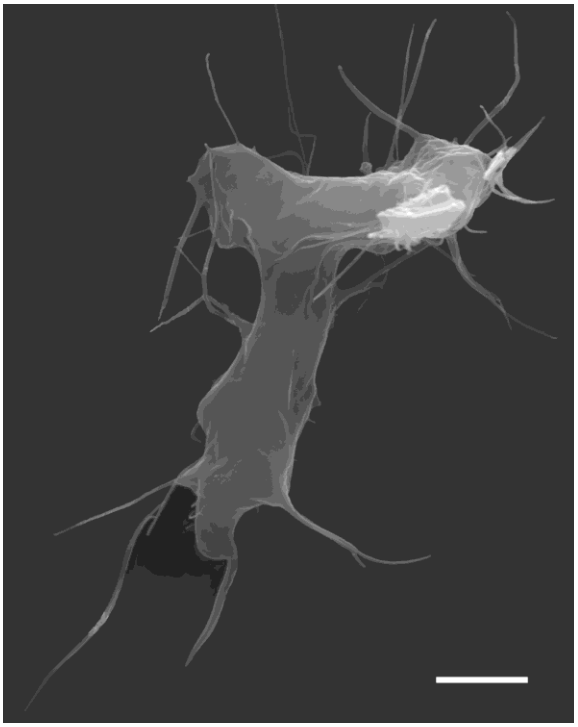

**FIGURE 1.** Scanning electron micrograph of a single IC isolated from guinea pig small mesenteric arteries. Scale bar: 5 µm.

cells, resulting from enzymatic dispersal of the tissue. Indeed, the transmission electron micrographs of artery segments did not show cells with excessive numbers of filopodia in the intact tissue, nor did these filopodia appear longer than the filopodia of isolated ICs[28,29,30] (although it can be argued that this observation is due to thinness of slices used in electron microscopy (~90 nm), which results in seeing only few filopodia in their incomplete length). Interestingly, when the cell isolation was performed in a culture medium (which has an abundance of nutrients), the resulting single ICs had fewer and shorter filopodia than the ones isolated in physiological solution containing only glucose as a nutrient[29]. This implies that nutrient deprivation (which would be the case if ICs were out of their natural environment) might stimulate the development of filopodia in ICs. A similar principle was found to operate in *Rhodnius* bugs, whose epidermal cells developed filopodia in response to oxygen deprivation[70]. Thus, the elongation of filopodia could be seen as a cell's effort to re-establish its connections. This logically leads to the question of whether the ICs in blood vessels are not merely an artefact of the cell isolation procedure – a result of the phenotypic modulation of VSMCs triggered by the disruption of tissue integrity during the isolation procedure and manifested, among other phenomena, by the development of filopodia (see below, in "Genuine cell type or artefact?").

The bodies of ICs are elongated and irregular (in contrast to spindle-shaped VSMCs). Only two exceptions have been observed so far: the stellate-shaped cells with filopodia described in the subendothelial intramuscular layer of rabbit portal vein[32] and at the external side of media in the human pulmonary vein[71]. Nothing is known about the function of this subpopulation of ICs, as their low abundance (in the case of rabbit portal vein) precluded more detailed investigation.

Approximately a third of arterial ICs which underwent time-lapse imaging during the investigation of their filopodia have shown an active change of their body shape[28]. This manifested as a generation or withdrawal of cytoplasmic protrusions at various positions on the cell body, giving it an irregular shape (Fig. 2). Similar to filopodial elongation, this process was also actin polymerisation–dependent and was taking place in a nutrient-poor environment with only glucose as a source of energy. In conjunction with

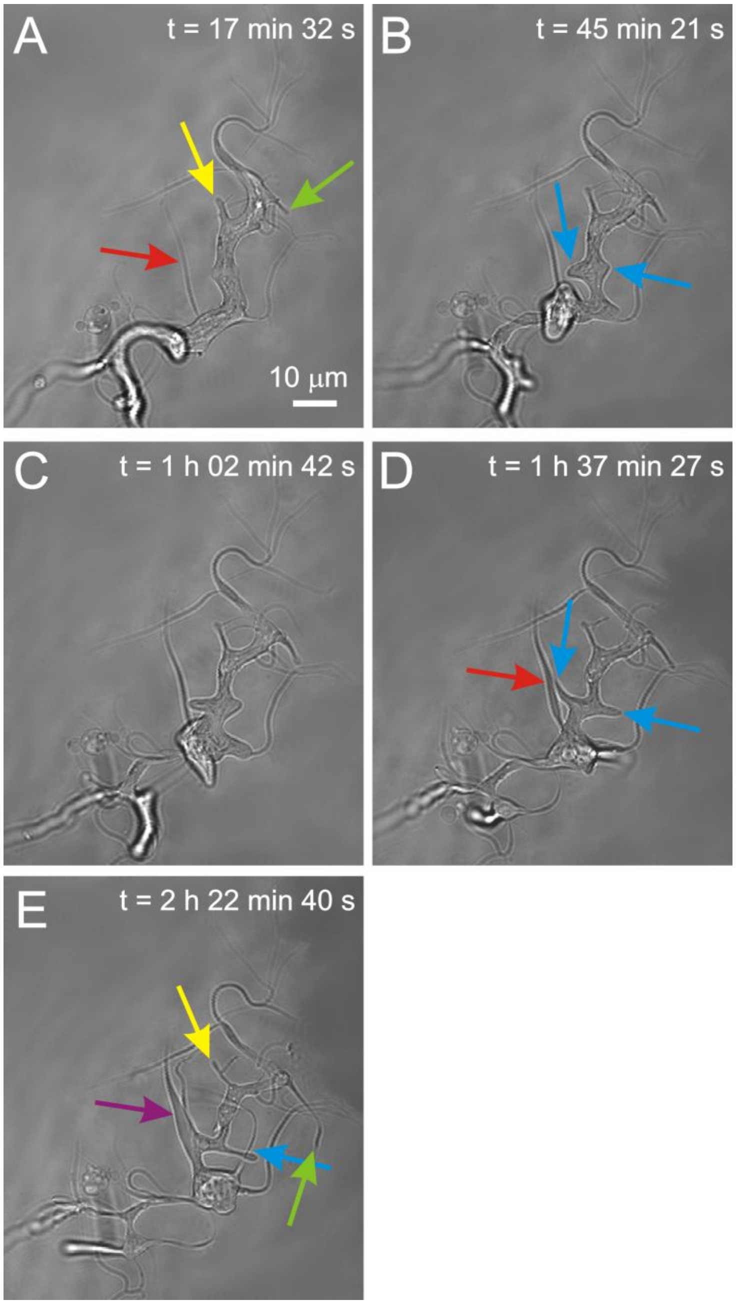

**FIGURE 2.** A series of transmitted light images of an arterial IC undergoing shape change. The cell was adhering to a laminin-coated coverslip and was placed in a physiological salt solution. Several different phenomena can be observed: elongation (green arrows), thinning (yellow arrows) or thickening of filopodia (red arrows), development of protuberations (blue arrows), and fusion of a protuberation with the thickened filopodium (purple arrow).

the fact that actin of vascular ICs (see below, in "Ultrastructure") is organised into stress fibres, this information suggested that they might be able to migrate, which gave rise to speculations that they participate in remodelling of the vascular wall. Such speculations are reinforced by the elongated shape of ICs, their localisation *in situ* among VSMCs, and the finding that morphologically similar cells, gastrointestinal ICCs, after having their c-kit receptors blocked with antibodies, dedifferentiate into a smooth muscle cell–like phenotype[72], all suggesting a close developmental relation between ICs and smooth muscle cells. However, there is currently no experimental evidence which would shed more light into the role of ICs in vascular remodelling.

## GENUINE CELL TYPE OR ARTEFACT?

The VSMC is regarded as a multifunctional mesenchymal cell[73] of substantial plasticity, capable of modulating its phenotype in response to changes in its microenvironment. It is therefore conceivable that severing its connections to the ECM and to adjoining VSMCs during a tissue-dispersal procedure could act as a trigger for the modulation of its phenotype. It was observed that upon placement of single isolated VSMCs in cell culture, they spontaneously and reversibly modulate their phenotype from "contractile" to "synthetic" within 3–7 days[74,75].

VSMCs are surrounded by basal lamina whose principal protein components are collagen IV and laminin. These proteins, bound to cells via integrins, transmembrane proteins capable of both inside-out and outside-in signalling[76], are known to promote the contractile VSMC phenotype[77,78]. It is the signals received through these contacts which appear to play a major role in maintaining mature contractile VSMCs in their current phenotype and preventing them from spontaneous phenotypic modulation into the synthetic phenotype, seen in cell cultures. Enzymes used for tissue dispersal, such as collagenase, elastase, or papain, cleave ECM proteins and could therefore damage or completely remove the basal lamina and expose the VSMCs to contacts with ECM proteins, such as collagen I or fibronectin (or their fragments), which promote VSMC conversion to a synthetic phenotype[77,79,80,81].

Significant efforts in the vascular IC research field were directed toward answering the question of whether they are genuine or their occurrence is triggered by tissue dispersion. There are several lines of indirect evidence which, observed as a whole, strongly suggest that ICs do reside constitutively in the vascular wall.

The strongest evidence is provided by the transmission electron micrographs of sections taken from intact vascular segments. These show cells with processes of similar thickness to those observed in single cell suspensions, located among VSMCs in the tunica media[28,29,30,31]. Using potassium permanganate as one of the staining steps resulted in differential staining of ICs (darker) compared to VSMCs[30].

Secondly, mature VSMCs would require a period of time to develop some of the ultrastructural features of ICs. The fact that ICs were found in single cell suspensions immediately after dissociation[28] makes it highly unlikely that mature VSMCs could, in such a short time, develop filopodia several tens of micrometers long, rearrange their actin cytoskeleton into stress fibres laying in various directions[28], or down-regulate smoothelin, a cytoskeleton-associated protein found only in contractile smooth muscle cells[82,83,84], which was all but absent in ICs of guinea pig small mesenteric arteries[29] (smoothelin has been reported to remain present in cells up to 5 days after the removal of tissue[82]).

Thirdly, if a supramaximal concentration of the actin polymerisation inhibitor latrunculin B is present continuously in the tissue from the moment of excision until its dissociation, it should block the development of any filopodia. If ICs were indeed an artefact of cell isolation, then only VSMCs would be seen in the resulting suspension. The fact that ICs were found in suspension despite this intervention[29] suggests that they must have existed in the tissue before the commencement of dispersion.

Fourthly, if the basal lamina was substantially corrupted or destroyed, the immunolabelling of its protein constituents in conjunction with confocal imaging would detect the damage. This was not the case – the cell surface coverage by laminin or collagen IV between the two cell types did not show substantial differences and the signal intensity in ICs was not lower either[29]. The experiments proved that ICs possessed basal lamina, which ruled out the possibility that they were myofibroblasts[85].

## ULTRASTRUCTURE

The ultrastructure of vascular ICs has not been investigated to such an extent as that of ICs in other tissues or of ICCs in the gastrointestinal tract, for which the ultrastructural criteria have been well established[60].

One of the first ultrastructural differences between vascular ICs and VSMCs was revealed not by electron, but by confocal microscopy. The actin staining patterns in ICs from guinea pig small mesenteric arteries produced by a fluorescent derivative of phalloidin have shown that actin was organised into stress fibres running in various directions in the cell. This is different to its arrangement in VSMCs, where it appeared tightly packed and no stress fibres could be seen[28], and probably reflects different functions of actin filaments in these two cell types. Two other cytoskeletal components, α-actinin and vinculin, also differed in their subcellular distribution, supporting observations on actin. Both of these proteins were distributed throughout the cytoplasm in arterial ICs, whereas in VSMCs, they were restricted to a thin subplasmalemmal layer[30]. While VSMCs require relatively fast sliding of actin and myosin filaments during contraction, the noncontractile (but not immobile) ICs could use actin stress fibres for the much slower type of movement during shape change and, presumably, migration.

Investigations using transmission electron microscopy did not reveal any dramatic ultrastructural differences between vascular ICs and the surrounding VSMCs. They have shown the presence of caveolae along the plasmalemma (in ICs of guinea pig small mesenteric arteries[28], human aorta[31], and rat cerebral artery[30]), multilobar nucleus[28,30,31], well-developed endoplasmic reticulum[30,31], intermediate filaments[31], and numerous mitochondria[30,31], mainly clustered around the nucleus. ICs formed cell-to-cell junctions with the adjoining VSMCs[29,30,31], but no gap junctions or specialised structures were detected in these zones in human aortic ICs[31]. In the filopodia of arterial ICs, the filaments could be sometimes seen embedded within the cytoplasmic matrix and running along the longitudinal axis of the filopodium, in parallel to the plasmalemma[28], but no other structures or varicosities were found.

Clearly, our knowledge of vascular IC ultrastructure is only at its beginnings. As the most prominent feature of ICs, the filopodia might hold important clues to IC function. For this reason, the investigation of their ultrastructure, especially in intact tissue, would be of high interest.

## LOCALISATION

ICs have so far been reported in rabbit portal vein[32], guinea pig small mesenteric arteries[28], rat middle cerebral artery[30], mouse mesenteric artery[86], human aorta and carotid artery[31], human pulmonary vein[71], and frog postcaval vein[34], with several other unpublished observations in various species and vascular beds. In the mesenteric artery tree, they were found in arteries of every branch order[29] (diameter range approximately 110–520 μm[28]). This observation and the presence of ICs in a range of vascular beds, and in species ranging from amphibian to several mammalian species, including humans, strongly argues in favour of the view that ICs accompany VSMCs throughout the body.

The information about the localisation of ICs in the vascular wall comes from transmission electron microscopy and, in the case of c-kit–positive ICs, from confocal microscopy. The localisation of ICs within the vascular wall varies and is dependent on the vascular bed. In rabbit portal vein and guinea pig mesenteric arteries, the ICs can be found both in the subendothelial layer and deeper in the media[28,29,32,35]; in rat cerebral artery, they are found deep in the media[30]; whereas in human aorta, they appear to be present along the media-adventitia border[31]; and in human pulmonary vein, on the external side and deep in the media[71]. In frog postcaval vein, a spontaneously active blood vessel, the methylene blue staining helped to visualise spindle-shaped elongated cells, occasionally with filopodia, located in the medial layer[34].

Worth noting is an irregular, cluster-like distribution of ICs described in rabbit portal vein[35] and in human aorta and pulmonary vein[31,71], which results in some vessel segments having a high density of

ICs and some sections having none. This is consistent with the existence of multiple, specialised pacemaker regions in the portal vein[87,88].

The works published so far did not find evidence that vascular ICs formed networks, unlike ICCs in the gastrointestinal tract[89]. Given their sparsity in the wall of guinea pig small mesenteric arteries (~5% of all cells in single cell suspensions[28]), their interconnection into networks seems unlikely if they are evenly distributed, as it would require filopodia hundreds of micrometers long. There are indications that rabbit portal vein ICs (which form clusters and are thus positioned closer to each other) are interconnected[35]. However, there are no data on the tissue level which would unambiguously support this hypothesis.

## MARKERS AND STRUCTURAL PROTEINS

The phenotype of a cell is defined by the proteins it expresses. Initial indications that ICs might be somehow related to smooth muscle cells were, in the case of vascular ICs, followed by investigations of proteins which these cells express and genes they transcribe. This was done in order to determine the cell lineage the ICs belong to, which could help with the elucidation of their physiological role(s), especially in arteries. Initial investigations focused on marker proteins for several cell types and have revealed that portal vein ICs were c-kit positive[32] and arterial ICs were not, but expressed smooth muscle myosin[28]. As the physiological role of portal vein ICs became clearer in the meantime, arterial ICs received more attention. Table 1 summarises the findings of experiments involving the immunocytochemical detection of proteins, and Table 2 lists the findings on gene transcription into mRNA obtained with quantitative or conventional reverse transcription PCR (RT-PCR).

**TABLE 1**
**Expression of Proteins by Vascular ICs and VSMCs**

| Protein Name | Expression in ICs | Expression in VSMCs |
|---|---|---|
| Protein gene product 9.5 | – [29] | – [29] |
| Von Willebrand factor | – [29] | – [29] |
| C-kit | + [32,86]; – [28,31] | – [28,32,86] |
| Desmin | + [28,30] | + [28,30] |
| Vimentin | + [28] | + [28] |
| Smooth muscle α-actin | + [29,30]; – [31,71] | + [29,30,71] |
| Smooth muscle myosin heavy chain | + [28,29,30] | + [28,29,30] |
| Smoothelin | (+)– [29] | + [29] |
| Myosin light chain kinase | (+)– [29] | + [29] |
| Calponin | – [30] | + [30] |
| Vinculin | + [30] | + [30] |
| α-Actinin | + [30] | + [30] |
| Fibronectin (on cell surface) | – [29] | – [29] |
| Collagen IV (on cell surface) | + [29] | + [29] |
| Laminin (on cell surface) | + [29] | + [29] |
| Methylene blue staining (whole tissue) | + [32]; – [28] | – [28,32] |

**TABLE 2**
**Transcription of Protein Genes by Vascular ICs and VSMCs**

| Gene for: | Transcription in ICs | Transcription in VSMCs |
|---|---|---|
| Protein gene product 9.5 | – [30] | – [30] |
| C-kit | – [30] | – [30] |
| Vascular endothelial growth factor A | – [30] | – [30] |
| CD34 | – [30] | – [30] |
| Prolyl-4-hydroxylase | – [30] | – [30] |
| CD68 | – [30] | – [30] |
| NG2 | – [30] | – [30] |
| Prominin 1 | – [30] | – [30] |
| Mast cell carboxypeptidase A | – [30] | – [30] |
| Desmin | + [114] | + [114] |
| Vimentin | + [114] | + [114] |
| Smooth muscle α-actin | + [114] | + [114] |
| Smooth muscle myosin heavy chain | + [30]<br>+ (SM1 isoform)[114]<br>– (SM2 isoform)[114] | + [30]<br>+ (SM1 isoform) [114]<br>+ (SM2 isoform)[114] |
| Myosin light chain kinase | + [114] | + [114] |
| Calponin | + (but ~4 times lower than in VSMCs)[30] | + [30] |

These results show that vascular ICs are negative for markers of neuronal cells (protein gene product 9.5[90]), endothelial cells (von Willebrand factor[91], vascular endothelial growth factor A[92], and CD34[93]), fibroblasts (CD34 and proline-4-hydroxylase[94]), macrophages (CD68[95]), pericytes (NG2[96]), stem cells (prominin 1[97]), or mast cells (mast cell carboxypeptidase A[98]). The expression of smooth muscle myosin heavy chain (SM-MHC) by ICs rules out the possibility that they are dendritic cells[99]. The absence of fibronectin and the presence of basal lamina proteins collagen IV and laminin on the cell surface[29], in conjunction with the expression of intermediate filament desmin in the cell cytoplasm[28,30], argue against ICs being myofibroblasts[85]. It is unlikely that ICs are fibroblasts as, apart from lacking fibroblast markers, they express smooth muscle α-actin and desmin, which are not expressed by fibroblasts[100]. The data in the tables corroborate the hypothesis that ICs belong to the VSMC lineage by demonstrating the presence of a number of proteins and smooth muscle markers (or their mRNAs) in both cell types (smooth muscle α-actin, SM-MHC, desmin, vimentin, vinculin, α-actinin)[101,102]. Other smooth muscle markers (smoothelin, myosin light chain kinase [MLCK], and calponin) were either absent or expressed significantly less in arterial ICs compared to VSMCs[29,30]. Because these markers are expressed by mature, differentiated smooth muscle cells capable of contraction, the arterial ICs, falling short of expressing the full protein inventory of VSMCs, appear to be their immature or dedifferentiated subpopulation.

Portal vein ICs were positive for c-kit, a tyrosine kinase receptor for stem cell factor, which is now an established marker of ICCs in the gastrointestinal tract[103,104]. C-kit labelling has replaced methylene blue staining as the principal method of visualisation of ICs. The presence of c-kit sometimes coincides with, but is not a requirement for, ICs' ability to produce rhythmic depolarisations, as demonstrated by c-kit–negative urethral ICs[23], and suggested by the ability of murine portal vein to produce rhythmic depolarisations and contractions despite having no c-kit–positive cells[105]. The disadvantage of both staining approaches is that they do not work in single isolated cells. It is presumed that c-kit is being shed and/or down-regulated from the cell membrane upon tissue dispersion.

Despite efforts, there is still no known specific marker for arterial ICs. This is perhaps the largest obstacle in this research field, as it precludes visualisation of ICs in intact tissues and separation of ICs from the bulk of VSMCs in single cell suspensions.

Vascular ICs did not contract after administration of noradrenaline, caffeine, membrane depolarisation by 60 mM $K^+$ solution, or cell membrane perforation[28,32], all of which reliably contracted VSMCs. As arterial ICs express both myosin and actin, it was initially unclear why they were noncontractile. Recent findings that the contraction-regulating proteins MLCK[29] and calponin[30] are down-regulated in arterial ICs at least partially explain this phenomenon. Myosin seems absent, for example, from rabbit urethral[23] or rat mesenteric ICs[50], and human aortic ICs did not express smooth muscle α-actin, but a portion of them expressed neurokinin 1 receptor strongly[31], all of which suggests heterogeneity of ICs.

## INTRACELLULAR CALCIUM

Information about changes in intracellular calcium comes from studies in ICs of rabbit portal vein[32,33,35] and guinea pig small mesenteric arteries[28].

The most obvious feature of *localised* intracellular calcium release is that, unlike in VSMCs, it is not restricted to subplasmalemmal regions[28,32], but can take place in the central cytoplasm or even filopodia. In filopodia, the transient localised increases in intracellular calcium concentration ($[Ca^{2+}]_i$), sometimes referred to as "$Ca^{2+}$ sparks", are asynchronous. This implies that they are locally controlled[32] and that there are calcium stores either within them or close to their roots (which is why ultrastructural studies of filopodia would be required).

Arterial ICs do not seem to be very active in terms of intracellular calcium release or changes in $[Ca^{2+}]_i$, as spontaneous calcium release events were produced in just over a quarter of them. The $Ca^{2+}$ sparks were mostly central and frequently resulted in the development of longer increases in $[Ca^{2+}]_i$, termed "calcium transients". Calcium transients varied in spatial spread, sometimes including a major portion and sometimes the whole of the cell volume, but none of them resulted in IC contraction and, if they were repetitive, they were irregular in rhythm[28]. Conversely, $Ca^{2+}$ sparks in VSMCs under these conditions remain localised and seldom develop into calcium transients[106,107]. This difference in spread might reflect the differences in spatial arrangement of intracellular calcium release microdomains between ICs and VSMCs and/or differences in their number or sensitivity. Changes in amplitude and duration of $Ca^{2+}$ signals were shown to control the activity of several transcriptional regulators differentially[108]. The spatiotemporal pattern of calcium signalling in arterial ICs is altered from brief and peripheral toward longer-lasting release events taking place closer to the nucleus. It is therefore possible that calcium release events in these cells not only regulate the activity of membrane proteins, but, in the longer term, also regulate/modulate gene expression.

Rabbit portal vein ICs displayed spontaneous rhythmical $[Ca^{2+}]_i$ oscillations at room temperature[32,33,35], which suggested their potential role of pacemakers. These repetitive calcium transients often developed from an initial localised $[Ca^{2+}]_i$ increase similar to a $Ca^{2+}$ spark[35], which originated from a discrete central region of the cytoplasm corresponding to a perinuclear sarcoplasmic reticulum rich in ryanodine receptors (RyRs)[33]. This is different from VSMCs, where $Ca^{2+}$ sparks originate from the subplasmalemmal sarcoplasmic reticulum[106,107].

The frequency of $[Ca^{2+}]_i$ oscillations in portal vein ICs was similar to their frequency of spontaneous membrane depolarisations and it was shown that the two coincided[33,35]. Investigation of the mechanism of $[Ca^{2+}]_i$ oscillations has shown that they resulted from $Ca^{2+}$ release from the sarcoplasmic reticulum through $IP_3$ receptors (IP3Rs) and RyRs, and that they depended on refilling of calcium stores through TRP nonselective cation channels, but not L-type $Ca^{2+}$ channels[33]. The dependence of calcium release in ICs on both IP3Rs and RyRs is reminiscent of VSMCs. There the initial $Ca^{2+}$ discharge is thought to be initiated by a RyR cluster and the IP3Rs support the propagation of $Ca^{2+}$ waves from one RyR domain to the other[109]. It is not known yet how the depolarisation occurs in portal vein ICs once

$Ca^{2+}$ ions reach the cell membrane. One possible mechanism is known from urethral ICs, where $Ca^{2+}$ ions activate calcium-dependent chloride channels resulting in $Cl^{-}$ efflux and depolarisation[55,110].

The frequency of $[Ca^{2+}]_i$ oscillations in rabbit portal vein ICs correlated with the rate of initial slow increase in $[Ca^{2+}]_i$ at the beginning of the increase. It seems that this phase determines the frequency of discharge and reflects some as-yet-unknown pacemaking process within the ICs[33]. However, only ~40% of portal vein ICs showed a clear slow increase phase, whereas in the other ~60% this phase was flat. The depletion of calcium stores did not seem to play a role in determining the frequency of $[Ca^{2+}]_i$ oscillations, as store refilling was fast (there was no refractory period) and happened during the descending phase of $Ca^{2+}$ wave[33].

In order to function as a pacemaker, a cell must be capable of not only producing rhythmic depolarisations, but also spreading them to other cells. For rabbit portal vein ICs, this proof came in the work of Harhun et al.[35]. Even following tissue dispersion, the ICs are sometimes seen contacting other cells (ICs or VSMCs) with their filopodia, offering a unique opportunity to investigate the transmission of signals between ICs themselves or between ICs and VSMCs. Technically difficult experiments involving confocal imaging of intracellular $Ca^{2+}$ and/or voltage-clamp of single cells have shown that the transmission of the calcium signal between two ICs was rapid (<200 msec), but the transmission between an IC and a VSMC was delayed by several seconds and did not involve spread of depolarisation through the filopodia. It was therefore hypothesised that a diffusible substance is released by ICs which, after reaching VSMCs, causes their depolarisation. While the identity of this substance is unknown for portal vein ICs, the work on urinary bladder ICs suggests prostaglandins as possible candidates[21].

## ION CHANNELS AND ELECTRICAL PROPERTIES

Apart from the higher capacitance of arterial ICs[28] presumably reflecting their bigger surface area, there were no significant differences in passive electrical properties between ICs and VSMCs[28,32].

The voltage-dependent membrane currents of ICs were similar to those of VSMCs[28,32]. Both cell types had a voltage-gated, nifedipine-sensitive, L-type $Ca^{2+}$ current and an outward current consisting of several, presumably $K^{+}$ current, components. In arterial ICs, there was little, if any, contribution of voltage-sensitive $Cl^{-}$ channels to the outward current[28]. The only difference between portal vein ICs and VSMCs was noticed in their spontaneous electrical events: while VSMCs discharged infrequent, but rapid, action potentials, the ICs produced repetitive, but slower, depolarisations[32](see above, in "Intracellular calcium"). Spontaneous transient outward currents (STOCs), indicative of stimulation of $Ca^{2+}$-activated $K^{+}$ channels by $Ca^{2+}$ sparks taking place in their vicinity, were seen in portal vein ICs. Arterial ICs, in contrast to VSMCs, produced no or very few STOCs[28] and this difference might reflect their different function. STOCs in VSMCs hyperpolarise the membrane and inhibit their contraction[111]. The role STOCs might play in noncontractile ICs is not clear as yet. It is possible that portal vein ICs, being able to generate rhythmic spontaneous depolarisations, need a powerful hyperpolarisation mechanism to modulate this activity, whereas arterial ICs, which do not seem to generate rhythmic depolarisations, have no need for such a mechanism.

The coupling of calcium release events to membrane currents in arterial ICs was generally weak (Fig. 3). Only occasionally long-lasting calcium release events coincided with slow outward currents, which was different to VSMCs, where brief and localised $Ca^{2+}$ sparks gave rise to similarly brief STOCs[28].

The membrane of portal vein ICs was labelled with anti-TRPC3,6,7 antibodies, suggesting the expression of at least one of these channel subunit types[33]. ICs did not have $Na^{+}$ current[28,32], further adding to the immunocytochemical and RT-PCR findings that they do not belong to neuronal lineage. The similarity in ion channel inventory between ICs and VSMCs argues in favour of the hypothesis that ICs belong to the smooth muscle cell lineage.

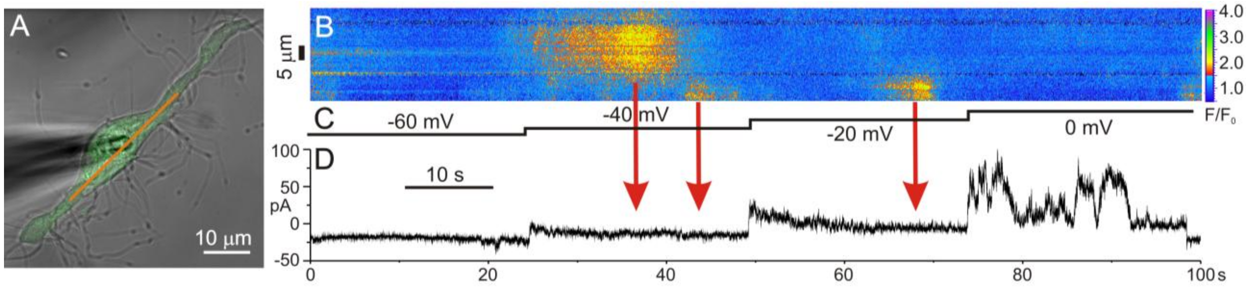

**FIGURE 3.** Loose/absent coupling of calcium release events and membrane currents in arterial ICs. (A) Transmitted light image of an arterial IC loaded with calcium-sensitive fluorescent indicator (green pseudocolour) and patched with a glass pipette. (B) Line scan image of normalised calcium fluorescence obtained by repeatedly scanning the orange line in panel A over 100 sec. X axis shows time, y axis shows the position on the scanned line. Fluorescence signal is colour-coded; pixels in yellow and red indicate elevated intracellular calcium concentration. (C) Voltage protocol imposed on the patched cell – its membrane was held at –60, –40, –20, and 0 mV, each step for 25 sec. (D) Membrane current of the patched cell, recorded simultaneously with calcium imaging. Small transient inward current can be seen at –60 mV and long-lasting transient outward current at 0 mV. Calcium release events in the cell did not elicit membrane current (red arrows).

## ROLE IN BLOOD VESSELS

The experimental data provide a strong case for pacemaking being the principal role of portal vein ICs. The presence of specialised pacemaking cells is consistent with the spontaneous activity of this vein[112]. These ICs produce spontaneous, rhythmical increases in $[Ca^{2+}]_i$[32,35], which can be transmitted to other cells either through direct contacts (in the case of IC-to-IC communication) or in a paracrine fashion (in the case of IC-to-VSMC communication)[35]. Moreover, rabbit portal vein ICs express c-kit[32], which is another feature they share with ICCs of the gastrointestinal tract. Little is known about any other markers the portal vein ICs might express and so it can be only assumed, by analogy to ICCs and arterial ICs, that they too belong to the smooth muscle cell lineage. The questions whether ICs in veins perform other roles, e.g., mediation of neural signals to VSMCs, or whether ICs from mammalian veins other than portal also act as pacemakers, await further experimentation. There are indications that pacemaking by ICs could be a widespread phenomenon. Data obtained on ICs of the urogenital tract[23,42,52] and on ICCs of the gastrointestinal tract[63], suggest that low-pressure tube systems, in general, need pacemakers to drive the vasomotion or peristalsis and help propel the tube contents (blood, urine, semen, chyme, and perhaps lymph) forward.

The above hypothesis is consistent with the observation that arterial ICs do not appear to act as pacemakers. In a high-pressure tube system, the forward movement of blood relies on the high-pressure gradient produced by the pumping action of the heart, so there is normally no need for spontaneous vasomotion and pacemakers. However, contraction waves coinciding with intercellular $Ca^{2+}$ waves, suggestive of vasomotion, were elicited by adrenoceptor stimulation in rat mesenteric artery strips[113]. It would be interesting to find out whether prolonged exposure of single arterial ICs to an adrenoceptor agonist results in rhythmic $[Ca^{2+}]_i$ oscillations.

Arterial ICs are found throughout the tunica media and transmission electron micrographs do not provide evidence of their proximity to nerve fibres[28,29,30]. For these reasons, their role as intermediaries between nerves and VSMCs does not seem likely.

Judging from their marker expression pattern, the arterial ICs belong to the smooth muscle cell lineage[29,30]. They express a majority of smooth muscle cell markers, but fall short of significantly expressing the proteins specific for the mature/contractile/differentiated phenotype: smoothelin, MLCK[29], calponin[30], and the SM2 isoform of SM-MHC[114]. This suggests that they are immature/phenotypically modulated/undifferentiated VSMCs. Although there is not enough evidence to call them “synthetic” VSMCs (as there is at present no proof that they migrate, synthesise ECM proteins, proliferate, and differentiate into contractile VSMCs), their functional properties (noncontractility, ability to change shape) indicate they might be just that. Constitutive presence of such cells in the vascular wall would be important for the maintenance of its integrity. VSMCs of synthetic phenotype can be regarded as oligopotent progenitor cells playing a central role in vascular repair, as they can migrate to the site of injury, synthesise ECM components, proliferate, and differentiate into the contractile phenotype to replace damaged cells[115]. This normally beneficial capacity can be harmful under some circumstances, as VSMCs of the synthetic phenotype have also been implicated in a number of pathological conditions stemming from disorders in vascular remodelling (e.g., atherosclerosis[116,117]; restenosis after angioplasty[115,118]).

Ideas that ICs might act as mechanoreceptors, sensing distension of the tissue they are in, or that they possess trophic or metabolic functions have been proposed on several occasions[28,53,119,120] (also in rat mesentery ICs[50]), but to date there is no experimental evidence to support them.

Thus, a picture emerges where vascular ICs are a part of the spectrum of smooth muscle cell phenotypes[53,121] ranging from ICCs specialised for pacemaking at one end to the mature smooth muscle cells specialised for contraction at the other (Fig. 4).

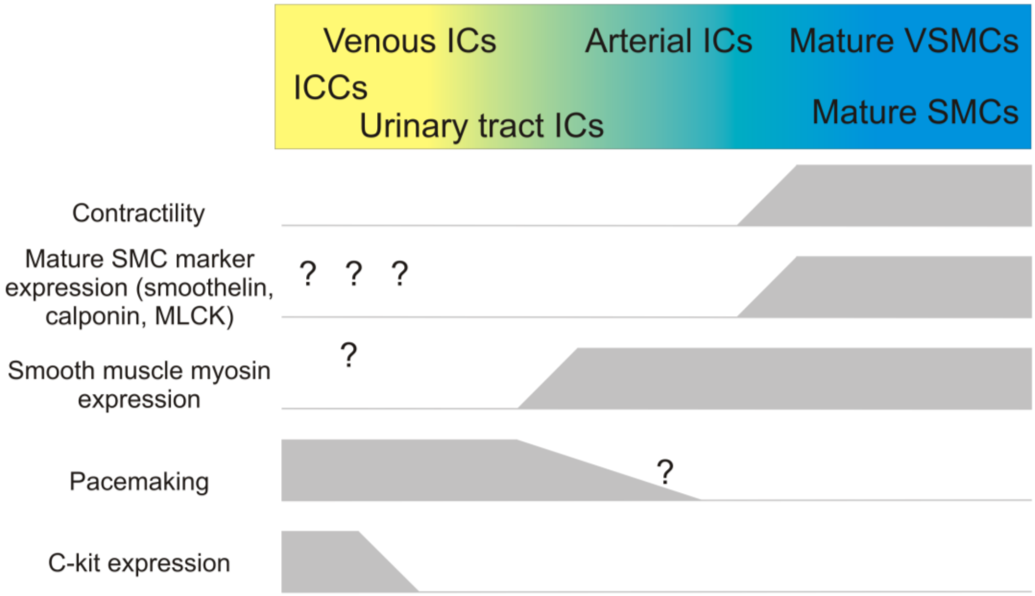

**FIGURE 4.** Diagram showing the possible spectrum of smooth muscle cell phenotypes and their features.

## FUTURE DIRECTIONS

Implication of vascular ICs in vasomotion and possible implication in vascular proliferative diseases, such as atherosclerosis, postangioplasty restenosis, or vein bypass graft failure[81], could make them a promising, albeit sparse, target for therapeutic interventions. The ability to modulate selectively the function of distinct VSMC subpopulations (ICs being one of them) could lead to new, advanced therapeutic approaches for a range of cardiovascular pathologies, which rank among the leading causes of morbidity and mortality globally. A detailed understanding of the function of VSMC subpopulations will be essential for their selective pharmacological targeting.

There have been only a handful of functional investigations of vascular ICs. Very little is known about ICs in some vascular beds, as the studies were carried out on a tissue level and only described the morphology of ICs. Future studies will have to address several important functional and structural aspects of vascular ICs.

The most pressing issue at the moment is the unavailability of a selective marker for arterial ICs. Such a marker would allow immunolabelling and visualisation of ICs in intact vessels, giving information about their number, localisation patterns, and possible connection into networks. A surface marker would boost investigations of single live arterial ICs by allowing their separation from the bulk of VSMCs in cell suspensions. The search for such a molecule using an immunocytochemical technique is slow and expensive, which is why high throughput (proteomic or molecular biological) approaches will be necessary. Such investigations would ideally result in a precise definition of various IC and smooth muscle cell subpopulations based on their protein inventories, which would clarify their position in the mesenchymal cell spectrum.

Functional experiments investigating whether arterial ICs are able to migrate, proliferate, and differentiate into mature VSMCs would be the most straightforward way to clarify whether they are indeed the VSMCs of synthetic phenotype. The different patterns of intracellular $Ca^{2+}$ release in ICs compared to VSMCs suggest regulation of different physiological processes. Better understanding of intracellular $Ca^{2+}$ signalling in arterial ICs will improve understanding of their function and may help to elucidate their role.

The portal vein is not a typical blood vessel. Its cellular arrangement with the outer longitudinal and the inner circular layer of smooth muscle cells is reminiscent of the intestine. It will therefore be important to establish whether there are ICs in other veins and, if so, whether they too act as pacemakers. The results obtained in the human pulmonary vein[71] and frog postcaval vein[34] indicate that both possibilities may be the case. Moreover, to better understand the origin and function of venous ICs, it would be interesting to establish whether they possess functional and structural features similar to arterial ICs: elongation of filopodia, active change of shape, expression of smooth muscle markers, and stress fibre–based actin cytoskeleton.

Lastly, the work indicating the presence of ICs in amphibian vein[34], suggests they are an evolutionarily old cell type. Extension of studies to amphibian arteries and to blood vessels of aquatic vertebrates would provide valuable data on the phylogenesis of ICs.

## ACKNOWLEDGEMENTS

The author would like to thank Prof. Alexander Zholos (QUB) for critical reading of the manuscript.
